# Draft genome sequence of *Priestia megaterium* strain IMGN3 derived from soil

**DOI:** 10.1128/mra.00458-24

**Published:** 2024-08-20

**Authors:** Sejin Choi, Hoseong Jung, Yeongjun Kim, Jeong A. Han, Eun Yu Kim, Ho-Seok Lee

**Affiliations:** 1 Center for Genome Engineering, Institute for Basic Science, Daejeon, South Korea; 2 Department of Biology, College of Sciences, Kyung Hee University, Seoul, South Korea; 3 Gyeonggido Agricultural Research & Extension Services, Hwaseong, South Korea; 4 Division of Natural and Applied Sciences, Duke Kunshan University, Kunshan, Jiangsu, China; 5 Environment Research Center, Duke Kunshan University, Kunshan, Jiangsu, China; University of Maryland School of Medicine, Baltimore, Maryland, USA

**Keywords:** *Priestia megaterium*, plant growth-promoting bacterium, biocontrol agent

## Abstract

*Priestia megaterium* sp. strain IMGN3 was isolated from the soil in South Korea. Here, we report its draft genome sequence, comprising 12 contigs with a total sequence length of 5.64 Mbp. This genome will provide valuable resources for future genomic studies, particularly focusing on plant growth promotion and biocontrol.

## ANNOUNCEMENT

*Priestia megaterium* (previously known as *Bacillus megaterium*) is an aerobic, rod-shaped, Gram-positive bacterium that adapts to diverse environments (1). In biotechnology, it has been researched as a potential host for industrial protein synthesis because of its high cloning host capacity and simple cultivation methods (2). Moreover, it is an ideal organism for environmental applications with its plant growth-promoting ability and potential as a biocontrol agent (BCA) (3), which promotes sustainable agriculture practices (4–6). This announcement will serve as a resource for comparative genomics, enhancing our comprehension of similar microbes and plant-microbiome interactions.

The new *P. megaterium* strain was isolated from a soil sample collected at a depth of 15 cm in the Yangpyeong Province (37.59707, 127.59836) of the Republic of Korea in 2019. The soil was diluted, spread onto TSA, and incubated for 16 h at 37°C. The culture was subsequently streaked two times under the same medium and conditions to obtain a single colony.

Genomic DNA was extracted using the Maxwell RSC Tissue DNA kit protocol and divided into two portions for different sequencing methods. One portion was sheared with the Megaruptor 3 (Diagenode) and purified using AMPure PB magnetic beads (Pacific Biosciences) for size selection. The PacBio sequencing library was prepared using the PacBio SMRTbell prep kit 3.0. HiFi sequencing was conducted on the PacBio Sequel II, yielding 92,291 reads. The mean length of HiFi reads was 9,077 bp, with an N50 value of 9,770 bp.

Second, the other portion was used to prepare the Illumina sequencing library using the TruSeq DNA Nano Library Prep Kit (Illumina, Inc., San Diego, CA, USA). Sequencing was performed on the Illumina HiSeq X Ten platform using a 2 × 150 paired-end protocol, generating total od 14,826,316 reads. Additionally, quality filtering and adaptor trimming were performed using Trimmomatic v0.38 (7), ensuring a phred score of 30 or higher in 90% of the bases.

The HiFi reads were employed for *de novo* assembly, performed using HGAP v4 (8) to generate the draft genome assembly, and the Illumina reads were used to refine and polish the genome assembly with Pilon v1.21 (9) three times. The final draft genome of IMGN3 comprised 12 contigs totaling 5,639,441 bp, with an average coverage of 148.4×, 37.8% GC content, and an N50 value of 5,137,220 bp. To assess assembly quality, BUSCO v5.1.3 (10) analysis indicated a high completeness of 99.19%.

Prokka v1.14.6 (11) was utilized to predict genes, resulting in 5,918 predicted genes, including 5,724 coding sequences, 146 tRNA genes, and 47 rRNA genes. Annotation was conducted using psiblast v2.7.1 (12) against the EggNOG DB v4.5 (13). The assembled draft genome was compared to the Genome Taxonomy Database (GTDB) via GTDB-Tk v2.3.2 (14) and assigned to *P. megaterium*. In each step, default parameters were used except where otherwise noted.

Genome annotation revealed functional genes for nitrogen fixation and production of beneficial compounds like polyamines, polyketides, and phenazine (15). These findings underscore the potential of this species in bioremediation and plant growth promotion.

## Data Availability

The annotated draft genome sequences of *P. megaterium* IMGN3 strain have been deposited at DDBJ/ENA/GenBank under the accession JBBLUG000000000. The version described in this paper is JBBLUG010000000. The raw reads are available under the BioProject accession number PRJNA1091902, with BioSample accession number SAMN40615794, and the Sequence Read Archive information for this project is SRR28604260 and SRR28604261.
